# Functionalized Electrospun Scaffold–Human-Muscle-Derived Stem Cell Construct Promotes In Vivo Neocartilage Formation

**DOI:** 10.3390/polym14122498

**Published:** 2022-06-19

**Authors:** Lina Jankauskaite, Mantas Malinauskas, Lauryna Aukstikalne, Lauryna Dabasinskaite, Augustinas Rimkunas, Tomas Mickevicius, Alius Pockevičius, Edvinas Krugly, Dainius Martuzevicius, Darius Ciuzas, Odeta Baniukaitiene, Arvydas Usas

**Affiliations:** 1 Institute of Physiology and Pharmacology, Lithuanian University of Health Sciences, LT-49264 Kaunas, Lithuania; mantas.malinauskas@lsmuni.lt (M.M.); lauryna.griniute@lsmuni.lt (L.A.); augustinas.rimkunas@lsmuni.lt (A.R.); tomas.mickevicius@kaunoklinikos.lt (T.M.); arvydas.usas@lsmuni.lt (A.U.); 2 Faculty of Chemical Technology, Kaunas University of Technology, LT-44029 Kaunas, Lithuania; lauryna.dabasinskaite@ktu.lt (L.D.); edvinas.krugly@ktu.lt (E.K.); dainius.martuzevicius@ktu.lt (D.M.); darius.ciuzas@ktu.lt (D.C.); odeta.baniukaitiene@ktu.lt (O.B.); 3 Pathology Center, Department of Veterinary Pathobiology, Veterinary Academy, Lithuanian University of Health Sciences, LT-47181 Kaunas, Lithuania; alius.pockevicius@lsmuni.lt

**Keywords:** cartilage regeneration, ozone-treatment, PCL scaffold, transforming growth factor beta3, human MDSCs, scaffold–cell construct

## Abstract

Polycaprolactone (PCL) is a non-cytotoxic, completely biodegradable biomaterial, ideal for cartilage tissue engineering. Despite drawbacks such as low hydrophilicity and lack of functional groups necessary for incorporating growth factors, it provides a proper environment for different cells, including stem cells. In our study, we aimed to improve properties of scaffolds for better cell adherence and cartilage regeneration. Thus, electrospun PCL–scaffolds were functionalized with ozone and loaded with TGF-β3. Together, human-muscle-derived stem cells (hMDSCs) were isolated and assessed for their phenotype and potential to differentiate into specific lineages. Then, hMDSCs were seeded on ozonated (O) and non-ozonated (“naïve” (NO)) scaffolds with or without protein and submitted for in vitro and in vivo experiments. In vitro studies showed that hMDSC and control cells (human chondrocyte) could be tracked for at least 14 days. We observed better proliferation of hMDSCs in O scaffolds compared to NO scaffolds from day 7 to day 28. Protein analysis revealed slightly higher expression of type II collagen (Coll2) on O scaffolds compared to NO on days 21 and 28. We detected more pronounced formation of glycosaminoglycans in the O scaffolds containing TGF-β3 and hMDSC compared to NO and scaffolds without TGF-β3 in in vivo animal experiments. Coll2-positive extracellular matrix was observed within O and NO scaffolds containing TGF-β3 and hMDSC for up to 8 weeks after implantation. These findings suggest that ozone-treated, TGF-β3-loaded scaffold with hMDSC is a promising tool in neocartilage formation.

## 1. Introduction

Articular cartilage is an essential tissue for structural and shape support, as well as friction on the joint head [1]. An increasing number of autoimmune-degenerative diseases, cartilage degeneration after traumatic injuries or due to aging is associated with loss of function, movement limitation, and decreased quality of life; therefore, it represents a high area of interest for a cartilage regenerative therapies. Due to avascularity and low density of cells, chondrocytes–cartilage is a complex subject in tissue regeneration [1,2]. To date, different methods for articular cartilage regeneration have been introduced into clinical practice without a clear effect and without ensuring long-term beneficial outcomes. Such methods as autologous chondrocyte transplantation or microfractures show contradicting results, and permanent recovery is doubtful [2,3,4,5,6]. Currently, tissue engineering focuses on the development of a biomimetic construct with a capability to recreate the structure and morphology of cartilage. Various materials and methods, with functionalization and/or loading of specific proteins, with or without additional cells, such as chondrocytes or stem cells, have been studied in vivo and in vitro [7,8,9,10,11,12]. Different techniques can be used to create the best environment for the cartilage regeneration. Several studies have revealed gas foaming, salt leaching, solvent casting, or 3D printing as effective techniques for scaffold formation [13,14,15]. Electrospinning, for instance, provides a possibility to generate loosely connected mattes with different thickness of continuous fibers [16]. Using natural and artificial polymers (e.g., PCL), a great structure mimicking extracellular matrix (ECM) regarding morphology and composition is created; thus, a proper close-to-physiological environment proper for different cells’ growth and proliferation is established [17]. Such material as polycaprolactone (PCL)—one of the most popular biomaterials in regeneration—is biodegradable, biocompatible, and non-cytotoxic, thus being highly suitable for application in neocartilage engineering [18]. In addition, loading of different functional groups via gas treatment (e.g., oxygen or nitrogen) has been shown to improve the properties of the scaffold and ameliorate cell attachment and growth [19,20,21]. Functionalization with transforming growth protein (TGF) β1 or TGF-β3, bone morphogenic proteins (BMPs), fibroblast growth factor (FGF) 2, and other proteins that are crucial for chondrocyte maintenance or stem cell differentiation into chondrocytes enhance scaffolds properties creating close to native counterpart [11,22]. Diverse cell–scaffold composites are studied for the best cartilage regeneration. Bone-marrow-derived or other mesenchymal stromal cells (MSC), muscle-derived stem cells (MDSCs), induced pluripotent stem cells (iPSC), or other stem cells have been used in articular cartilage regeneration process [4,23,24]. Chondrocyte–scaffold constructs are being investigated as well [25]. Nevertheless, various seeding densities, cell phenotypes, or passages can result in distinct sometimes contradictory results [26].

In our study, we aimed to create an improved cell–scaffold construct for cartilage regeneration first to test its efficiency in vitro and then for in vivo animal application. In this article, we present hMDSCs inoculated into ozone-treated transforming growth factor (TGF) β3 functionalized PCL scaffold. We investigated scaffold’s potential to provide and maintain a microenvironment for hMDSCs migration, proliferation, differentiation into chondrocytes, and supporting cartilage-like tissue regeneration in vitro and in an in vivo mouse model. On the basis of the obtained results, we believe our findings will significantly contribute to the cartilage regeneration field.

## 2. Materials and Methods

### 2.1. Scaffold Fabrication, Functionalization, and Protein Binding

Poly(e-caprolactone) (PCL) scaffolds were prepared as described previously [27]. Briefly, the scaffolds were fabricated of PCL fibers with the addition of cellulose acetate using the custom-made solution cryo-electrospinning setup [28]. The scaffolds were post-treated to convert cellulose acetate to cellulose and to introduce functional groups for the binding of the growth factor [27]. The fabricated fibrous mats were cut into 0.5 g specimens and subjected to the solution of 0.05 M NaOH/water-ethanol at 25 °C for 24 h. Afterwards, the scaffolds were placed in a glass reactor containing water (20 ± 1 °C) and treated by bubbling O_3_ from an ex situ generator (GL-3188A, Shenzhen Guanglei, Shenzhen, China) into the reactor at a mass flow of 400 mg/h. Subsequently, the samples were stored in a vacuum dryer at 21 ± 2 °C for 12 h. The morphology, chemical composition, hydrophilicity, absorption, and thermal and mechanical properties of the scaffold were evaluated and described in previous research [27]. The scaffold featured a random fibrous structure with a mean fiber diameter of 9.3 ± 4.1 μm, and a pore size ranging from 50 to 300 μm, having an overall scaffold porosity of 90.7%. The presence of the main components (PCL and CA) and their transitions (CA to CEL) were confirmed by FTIR, as the intensity of the 1287 cm^−1^ peak (C-O and C-C stretching) induced due to introduced carboxyl groups confirmed the presence of functional groups after treatment. Each of the treatment stages brought in a significant change in wettability and resulted in 72.4 ± 7.5° water contact angle. Such range is considered superior to the cell proliferation as opposed to the intrinsic hydrophobicity of PCL. After functionalization, fibrous mats were cut into scaffold specimens of 6 mm in diameter, 1.5 mm in height, and weight of 0.0056 ± 0.0002 g, then sterilized with ethylene oxide and further used for in vitro and in vivo experiments. After functionalization, PCL scaffolds were incubated with 10 ng/mL TGF-β3 (Thermo Fisher Scientific, Waltham, MA, USA). First, the efficiency of protein binding with the “naïve” (NO) or ozone-treated (O) scaffold was tested after incubation with TGF-β3 for 0.5, 1, 2, 4, 8, and 24 h. Afterwards, wash-out of unbound protein and testing via ELISA were performed. Finally, for in vitro and in vivo experiments, samples were covered with 100 μL of 10 ng/mL TGF-β3 for 24 h. Subsequently, the unbound protein was washed with PBS, and scaffolds were submitted for further experiments.

### 2.2. Human Cell Cultures

For human-muscle-derived stem cell (hMDSC) and human chondrocyte (hCH) isolation, the ethical consent was approved by the Regional Medical Ethics Committee (No. BE-2-22); all patients provided their written consent for biopsy material to be used in laboratory research. Six patients (2 female, 4 male) with a mean age of 30 years [18,19,20,21,22,23,24,25,26,27,28,29,30,31,32,33,34,35,36,37,38,39,40] underwent an anterior cruciate ligament reconstruction (ACL reconstruction).

### 2.3. hMDSC Isolation and Monoculture

hMDSCs were isolated from adult skeletal muscle using pre-plating technique with some modifications [29]. After washing; dissecting from residual tendon, fat, and connective tissue; and mechanical digestion, minced muscle tissue was submitted for enzymatic digestion with 0.2% of collagenase type XI and kept with gentle continuous rocking at 37 °C for 1.5 h. Further, the previously described protocol was followed with an adjusted centrifugation speed of 1.400 rpm for 5 min [29]. Obtained cells were cultivated in monolayer on a collagen-coated surface using Dulbecco’s modified Eagle’s medium (DMEM) with 4.5 g glucose/L (Gibco, UK), supplemented with 10% fetal bovine serum (FBS) (Gibco, UK), 10% horse serum (HS) (Gibco, UK), 0.5% chicken embryo extract (LSP, UK), and 1% penicillin/streptomycin (penicillin/streptomycin (10,000 U/L) (P/S), Gibco, UK) at 37 °C, 5% CO_2_, in a humidified incubator. The medium for hMDSCs was changed every 3 days. Passage 5 to 10 were used for further experiments.

First, the morphology of cells was evaluated via light microscopy. The lineage of isolated hMDSCs was identified by flow cytometry for strain biomarkers and by differentiation ability. Third passage of hMDSCs was tested for CD45, CD34, CD59, CD90, and CD117 (Invitrogen, Waltham, MA, USA) using flow cytometry (FACSMelody (BD)). Multipotent differentiation capacity was proven by induced adipogenic, and osteogenic differentiation in monolayer, and chondrogenic differentiation in pellet culture. Adipogenesis was induced with an adipogenesis differentiation medium (Gibco, UK). After 14 days of cultivation, adipogenic cultures were stained with Oil Red O (Sigma-Aldrich, St. Louis, MO, USA) for microscopy visualization of lipid droplets. Osteogenesis was evaluated via microscopy when cells were stained with Alizarin Red (Sigma-Aldrich, USA) after 14 days of incubation with osteogenesis differentiation medium (Gibco, UK). Chondrogenic capacity was proven by successful chondrogenic pellet formation after 21 days of incubation in chondrogenesis differentiation medium (Gibco, UK) and stained with Safranin O (Sigma-Aldrich, USA) and Toluidine Blue (Sigma-Aldrich, USA). After differentiation, hMDSCs were fixed with 1% PFA and stained as previously described. Fixed differentiated hMDSCs were evaluated via microscopy (Olympus BX63, Tokyo, Japan). 

### 2.4. Human Chondrocyte Isolation and Monoculture

Each biopsy sample of articular cartilage was taken from the intercondylar notch and immersed in a 50 mL conical sterile polypropylene centrifuge tube (TPP, Trasadingen, Switzerland) containing a total of 15 mL transport medium with 12 mL of DMEM, 3 mL of fetal bovine serum (FBS) (Gibco, UK), and 15 µL of 0.1% gentamycin (Gibco, UK). The biopsy was transported immediately to the laboratory for a further chondrocyte isolation procedure. The sample of articular cartilage was washed three times with Hams/F12 (Gibco, UK) containing 1% penicillin/streptomycin (penicillin/streptomycin (10,000 U/L), Gibco, UK). The cartilage was then minced with a sterile scalpel blade into small pieces in a Petri dish containing 2 mL of proteases (type XIV, >3.5 U/mg, Sigma-Aldrich, USA) solution. The minced cartilage was transferred into a tube with 8 mL of protease solution and left for digestion for 60 min at 37 °C, 5% CO_2_. After 60 min, the proteases solution was changed to 10 mL of collagenases (type A > 150 U/mg, Worthington Biochemical, Lakewood, NJ, USA) solution, and cartilage pieces were digested for 16 h at 37 °C, 5% CO_2_. The enzymatic reaction was neutralized with 10 mL of DMEM supplemented with 10% FBS and 0.1% gentamycin (medium) and then filtered through a 70 µm cell strainer (Falcon, UK) into a 50 mL tube and centrifuged at 300× *g* (4 °C) for 10 min. The supernatant was carefully discarded, and the cell pellet was filled with a fresh 1 mL of medium. The mixture was resuspended, and the cell count also the viability was determined by trypan blue dye exclusion test. The cells were plated in tissue culture flasks at a density of 2 × 10^3^ cells/cm^2^. The morphology of the cells was examined regularly viamicroscope. When the cells reached confluency of 80% to 90%, they were trypsinized with TrypLE-Express enzyme (1×), phenol red (Gibco, UK). Harvested chondrocytes (passage 0 (P0)) were further centrifuged and resuspended in a culture medium. Culture medium was changed every 3 days. Cells were replated at a density of 2–6 × 10^3^ cells/cm^2^. 

### 2.5. Pellet Culture

hMDSC and chondrocyte (hCH) pellets were established in microcentrifuge tubes by suspension of 2 × 10^5^ cells in 400 µL of culture medium. The tubes were centrifuged at 300× *g* (4 °C) for 6 min. The tops of the tubes were perforated with 18-gauge needle after centrifugation to permit gaseous exchange. After 72 h, corresponding to the first time the medium was changed, the pellets were gently detached from the bottom. This procedure was repeated every 3 days until the 21 days pellet culturing time point was reached. The hMDSCs pellet was cultured in a chondrogenesis differentiation medium (Gibco, UK). Control pellet containing hMDSC cells were cultured using an identical cell culture medium described in the section of hMDSC isolation and monoculture. The triplets of both pellets including hMDSC and control were formed.

### 2.6. Cell-Scaffold Culture In Vitro and Cell Tracking

The cell–scaffold constructs were divided into the following groups: control group (cells on NO or O) and cells on scaffolds loaded with TGF-β3 (NO + TGF-β3 or O + TGF-β3). hCH or hMDSCs were seeded at the density of 50,000 cells per scaffold. Ten microliters of cell suspension were seeded on each scaffold and incubated for 1 h at 37 °C, 5% CO_2_. Further, an additional 90 μL of the medium was added, and all samples were cultured in a culture medium at 37 °C, 5% CO_2_. 

For cell tracking experiments, hCH or hMDSC were stained with a pkh-26 red fluorescent cell linker kit (Sigma-Aldrich, USA) according to the manufacturer’s instructions. Briefly, cells were centrifuged, and 1 mL (for 2 × 10^7^ cells) of Diluent C was added, followed by 1 mL of 2× Dye Solution, and the mixture was incubated for 1–5 min. The staining was stopped with a 1% bovine serum albumin (BSA) solution and incubated for an additional minute. Cells were washed twice and resuspended in medium for further tracking experiments. Stained hCH or hMDSCs were seeded on scaffolds and followed for 21 d. Cell–scaffold constructs were inspected via microscopy and evaluated on days 1, 3, 7, and 14.

### 2.7. Cell Proliferation Assay

Unstained cell–scaffold complexes were cultivated in 96-well plates. Briefly, cells were seeded on scaffolds at a density of 50,000 cells per scaffold. Three replicates were set up for each group. Cell proliferation was examined at the following time points: days 1, 3, 7, 14, 21, and 28. On each day, 10 μL of the CCK-8 (ab228554) solution was added to each well with the cell–scaffold complex. After 3–4 h incubation, the medium was collected into a separate 96-well plate, and the absorbance of each well was measured using a microplate reader (Multiskan GO 1.00.40 (Thermo Fisher Scientific)).

### 2.8. Enzyme-Linked Immunosorbent Assay (ELISA)

TGFβ-3 and collagen 2 (Coll2) concentrations in the supernatants were quantified using ELISA kits. In the cell culture medium from TGFβ-3-loaded and “naïve” NO or O and cell complexes, both human MDSC and hCH were aspirated on days 1, 3, 7, 14, 21, and 28; frozen at −80 °C; and kept until needed (less than one month). TGFβ-3 and Coll2 were measured according to the protocols of commercially available ELISA kits: TGFβ-3 (Assay Biotech, Fremont, CA, USA) and Coll2 (Cloud-Clone Corp., Katy, TX, USA). Absorption at 450 nm was measured using a microplate reader (Multiskan GO 1.00.40 (Thermo Fisher Scientific)).

### 2.9. In Vivo Animal Experiments

**Figure 1:**
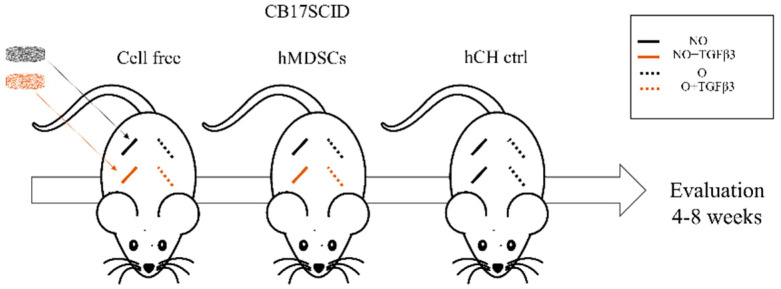
Experimental in vivo mouse model. Different scaffolds—NO and O with or without a growth factor (TGFβ-3) and with or without hMDSCs, and hCH as control were implanted into CB17SCID mice, and mice were followed up for 4 and 8 weeks. hMDSCs—human-muscle-derived stem cells; hCH—human chondrocytes; ctrl—control; NO—not ozonated; NO- TGFβ-3—not ozonated, treated with TGFβ-3; O—ozonated; O- TGFβ-3—ozonated, treated with TGFβ-3; TGFβ-3—transforming growth factor beta-3.

All in vivo experiments were performed in accordance with the permission from the ethics committee of the State Food and Veterinary Service (No G2-133). Fourteen CB17SCID female mice 6–7 weeks of age purchased from Animalab (Riga, Latvia) were used for the subcutaneous implantation of the scaffolds (Figure 1). NO and O scaffolds were assigned to different groups on the basis of the way they were prepared. There were cell-free or hMDSC containing NO or O scaffolds that were with TGFβ3 (10 ng/mL) or growth factor free. Growth-factor-free NO and O scaffolds with hCH were used for positive control. Under general isoflurane anesthesia, four separate horizontal skin incisions were made in the back of each mouse. Scaffolds from different groups were implanted in the subcutaneous pockets created in the back of each mouse (four scaffolds per mouse). After 4 and 8 weeks, mice were sacrificed; the scaffolds were removed and fixed in 4% paraformaldehyde (PFA) (Sigma-Aldrich, USA).

### 2.10. Histology and Microscopy

Samples from in vivo experiments were embedded in paraffin and sectioned at the thickness of 8 µm. Safranin O staining was performed using standard staining protocol. Stained cells or images of the stained sections were obtained using a light microscope (Olympus BX63, Japan) equipped with Olympus DP72 camera. Images were processed for histomorphometry analysis and assessed for the red color intensity using image-processing software (Cell-Sens Dimension Olympus Soft Imagine Solutions, Muenster, Germany). The histological assessment was performed by two independent investigators that were blinded to which group sample belongs. Four different areas from the in vivo section of each sample were analyzed, and the percentage of average Safranin-O-positive area was determined.

### 2.11. Coll2 Immunohistochemistry

Sections were deparaffinized, retrieved with pepsin, and blocked with hydrogen peroxide for 10 min. After washing and protein blockade, sections were incubated overnight at 4 °C with primary anti-collagen II antibody (Abcam 185430). After washing with buffer solution, a secondary biotinylated goat-anti-mouse IgG antibody (Abcam 64255) was applied for 10 min. After application of streptavidin peroxidase for 10 min and washing with PBS buffer, sections were incubated with DAB, washed with distilled water, and counterstained with hematoxylin.

### 2.12. Statistical Analysis

Statistics were accomplished using IBM SPSS 28.0 software (SPSS Inc., Chicago, IL, USA) for Windows. All the quantitative data were expressed as mean +/− standard deviation (SD). Mann–Whitney nonparametric independent sample test was used for statistical analysis of red color intensity measurement average. One-way ANOVA and Tukey’s post hoc test were used. A *p*-value < 0.05 was set as significant.

## 3. Results

### 3.1. TGF-β3 Binding to Ozone-Treated or Untreated PCL Scaffolds

**Figure 2:**
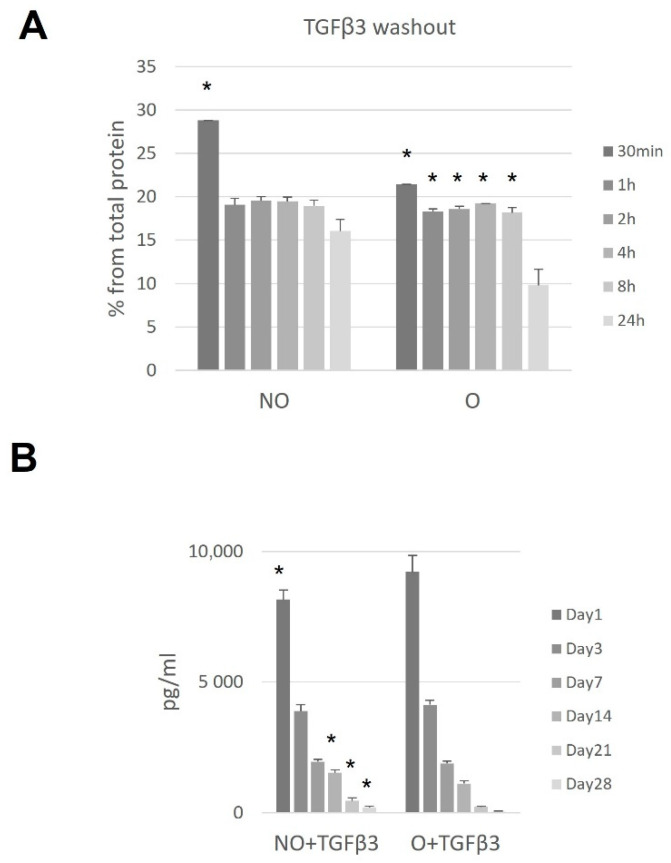
TGF-β3 wash-out (**A**); TGF-β3 release within time (**B**). Scaffolds were loaded with TGF-β3 and tested for protein binding (n = 2) and release (n = 5) at the given timepoints. Results are presented as mean +/− standard deviation. The * represent *p*-value < 0.05 comparing values after 24 h as a reference level (**A**) and comparing NO + TGF-β3 at all timepoints to O + TGF-β3 scaffolds (**B**). NO—untreated, O—ozone-treated, TGF-β3—transforming growth factor beta-3, min—minutes, h—hours, pg/mL—picograms per milliliter.

Prior to any experiments, possible TGF-β3 binding on NO or O scaffolds was evaluated. ELISA measurements of the wash-out revealed best binding after incubating for 24 h with the significantly better protein-trapping on ozonated scaffolds (30 min compared to 24 h *p* = 0.017 as for O, and *p* = 0.0056 as for NO) (Figure 2A). Both scaffolds demonstrated constant protein release with a significant decrease within time (Figure 2B). Ozone-treated scaffolds proved better binding capacity and less release within 1 day and after 14, 21, and 28 days (Figure 2B).

### 3.2. hMDSC Profile

**Figure 3:**
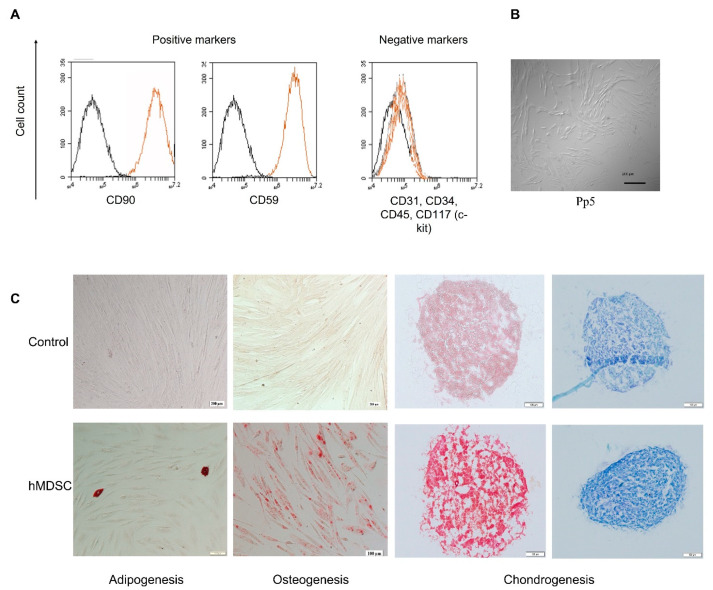
Characterization of isolated hMDSCs. The expression of specific stem cell markers was tested via FACS (**A**) and differentiated into adipocytes, osteocytes, and chondrocytes (**C**). Isolated cell (preplate 5, passage 5) morphology was identified via microscopy (**B**). A—black color—negative control, orange—stained; C—adipogenesis—Oil Red O staining; osteogenesis—Alizarin staining; chondrogenesis—safranin (orange-red) and toluidine blue (blue) staining. CD—a cluster of difference; pp5—preplate 5; hMDSC—human-muscle-derived stem cells. Scale bar—200 μm.

Human MDSCs were isolated from a few donors and analyzed by flow cytometry (FACS). FACS analysis showed the expression of stem cell surface markers (CD59 and CD90) and lack of hematopoietic markers (CD34, CD45). However, they lacked to express CD117 (Figure 3A). Moreover, hMDSC showed the multipotent capacity to differentiate into adipogenic, osteogenic and chondrogenic cells (Figure 3C). Multilineage differentiation capability confirmed the origin and stem-cell-specific properties of isolated cells (Figure 3).

### 3.3. PCL Scaffold—hMDSC Construct

**Figure 4:**
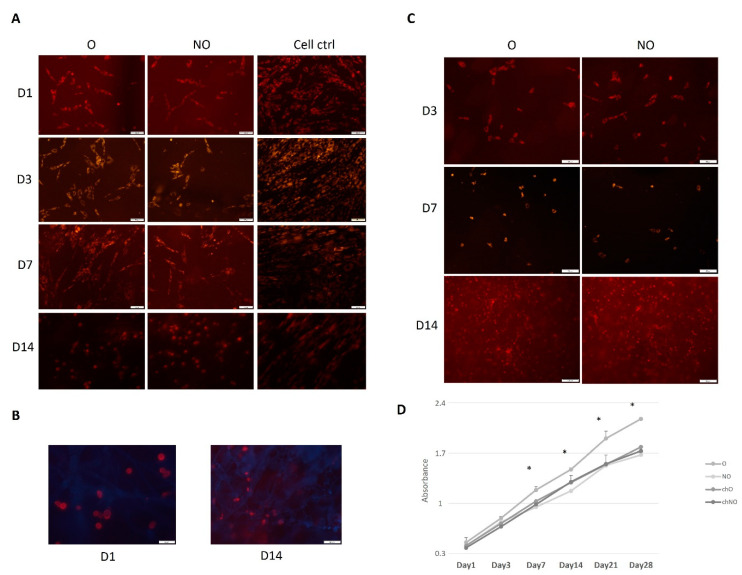
hMDSCs and hCH tracking (**A**–**C**) and proliferation (**D**) on “naïve” and ozone-treated PCL scaffolds. hMDSC and hCH were pkh-26-stained and followed for 14 days (**A**–**C**), blue color (**B**)—stained with DAPI. Unstained cells were seeded, and cell proliferation was evaluated via CCK-8 until day 28. Results are presented as mean +/− standard deviation. * represents *p*-value < 0.05. NO—untreated, O—ozone-treated, chNO—untreated scaffolds with chondrocytes, chO—ozone-treated scaffolds with chondrocytes, ctrl—control, D—day. Scale bar—100 μm (**A**,**C**) and 50 μm (**B**).

The pkh-26-stained hMDSCs or hCH (as control) were seeded and followed on the NO and O scaffolds for 14 days. hMDSCs showed successful spreading already within one day after seeding (Figure 4B) and specific cell morphology in both scaffolds compared to control cells (hMDSC seeded on plastic) (Figure 4A). The same was observed with chondrocytes (Figure 4C). On day 14, cell morphology was unable to be identified due to disintegration of the scaffold. All the cells were proliferating similarly until day 7, when hMDSC began to proliferate better in O scaffolds compared to NO and hCH within both types of the scaffolds, having significantly superior proliferation rates at days 14, 21, and 28 (*p* < 0.01).

### 3.4. Ozone-Treated TGF-β3 Containing Scaffold—hMDSC Construct

**Figure 5:**
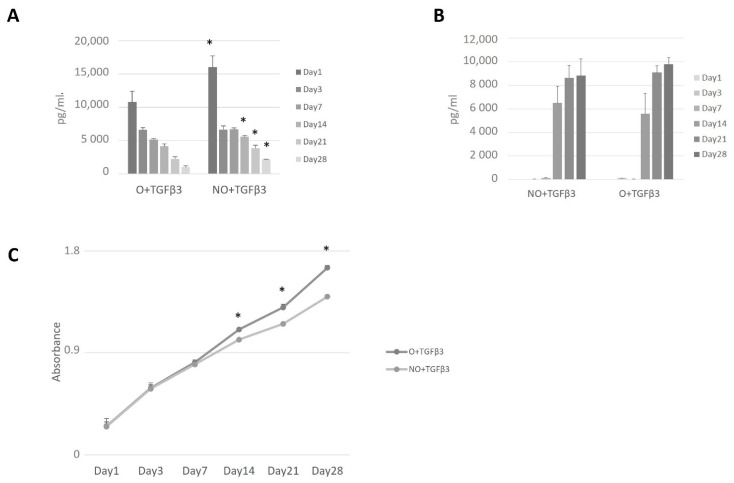
TGF-β3 release from the scaffold with hMDSCs (**A**), collagen production from ozone-treated and untreated scaffolds (**B**), and cell proliferation within functionalized scaffolds (**C**). TGF-β3 and collagen-II protein levels were tested at days 1, 3, 7, 14, 21, and 28 via ELISA. Cell proliferation rate was tested at the given timepoints via CCK-8. Results are presented as mean +/− standard deviation. * represents *p*-value < 0.05 comparing NO + TGF-β3 at all timepoints to ozonated scaffolds (**A**,**B**). NO—untreated, O—ozone-treated, TGF-β3—transforming growth factor beta-3, pg/mL—picograms per milliliter.

Comparing both types of PCL scaffolds, we observed significantly higher amounts of released TGF-β3 from NO scaffolds (Figure 5A). We tested untreated scaffolds to assess the hMDSCs production of TGF-β3; however, no protein was identified (data not shown). After scaffold protein treatment, higher proliferation was observed on O_3_-treated PCL scaffolds, and from day 14, the proliferation rate was higher compared to untreated scaffolds (Figure 5C). Nevertheless, it did not reach the growth rate on “naïve” scaffolds (Figure 5D).

Coll2 protein expression was observed from day 14 with increasing quantities. Slightly higher Coll2 levels were found on days 21 and 24 within supernatants of O_3_-treated, TGF-β3 loaded PCL scaffolds; however, a significant difference was not determined (Figure 5B).

### 3.5. Enhanced Formation of Safranin-O-Positive Matrix on TGF-β3- and hMDSC-Containing Scaffolds

**Figure 6:**
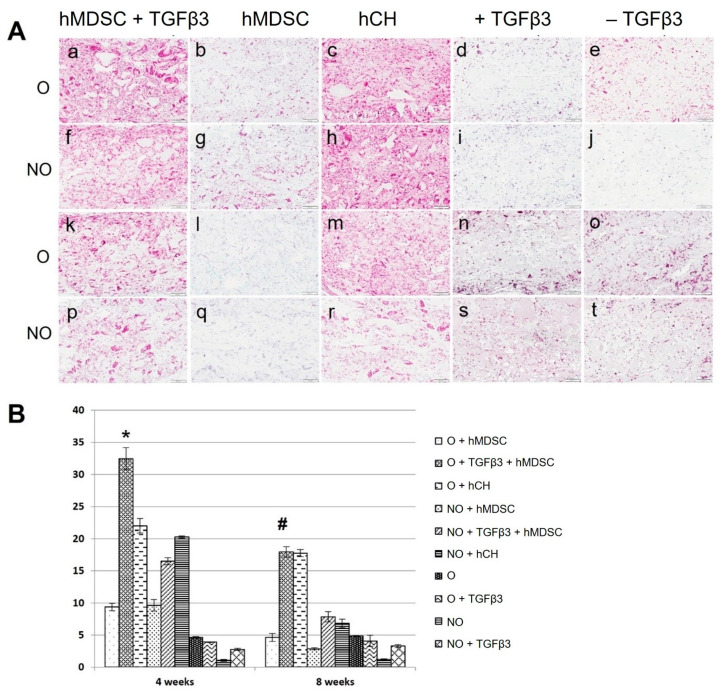
GAG formation in O and NO scaffolds in vivo after 4 weeks (a–j) and 8 weeks (k–t) (**A**), and quantification of Safranin O staining (**B**). Cell-free scaffolds (d,e,I,j,n,o,s,t). NO—untreated, O—ozone-treated, hMDSC—human-muscle-derived stem cells, hCH—human chondrocytes, TGFβ3—transforming growth factor beta-3. Results are presented as mean +/− standard deviation. The * represents *p*-value < 0.05 compared to all other treatment groups after 4 weeks. The # represents *p*-value < 0.05 compared to all other treatment groups except the O + hCH group at 8 weeks. Scale bar—100 μm.

Safranin O staining after 4 and 8 weeks revealed diverse formation of glycosaminoglycans’ (GAG) positive matrix within the implants. O and NO scaffolds loaded with TGFβ3 and seeded with hMDSCs demonstrated a more pronounced manifestation of Safranin O positive areas compared to hMDSC–scaffold constructs without TGFβ3 (Figure 6A). hCH–scaffold constructs also showed more abundant Safranin-O-positive staining after 4 weeks, which slightly diminished after 8 weeks. There was no obvious manifestation of GAGs in cell-free O and NO scaffolds (control group) in the presence or absence of TGFβ3 at any time (Figure 6A).

Quantification of GAG deposition after 4 weeks revealed a significant increase in Safranin-O-positive area in O scaffolds containing TGF-β3 and hMDSCs when compared to the same scaffolds without TGF-β3 or with similar groups of NO scaffolds (Figure 6B). There was no significant difference between O and NO scaffold groups with hCH at week 4, but the average positive area was significantly lower compared to the O scaffold group containing TGF-β3 and human MDSCs. Safranin-O-positive area in control cell-free scaffolds was barely detectable.

After 8 weeks, the Safranin-O-positive area decreased in both O and NO scaffold groups containing hMDSCs but remained significantly higher in scaffolds loaded with TGF-β3 compared to scaffolds without protein. There was no difference between O scaffolds populated with hCH and O scaffolds containing TGF-β3 and hMDSCs; however, the Safranin-O-positive area was significantly smaller in the NO scaffold group with hCH. The Safranin-O-positive area in control cell-free scaffolds after 8 weeks remained barely detectable (Figure 6B).

**Figure 7:**
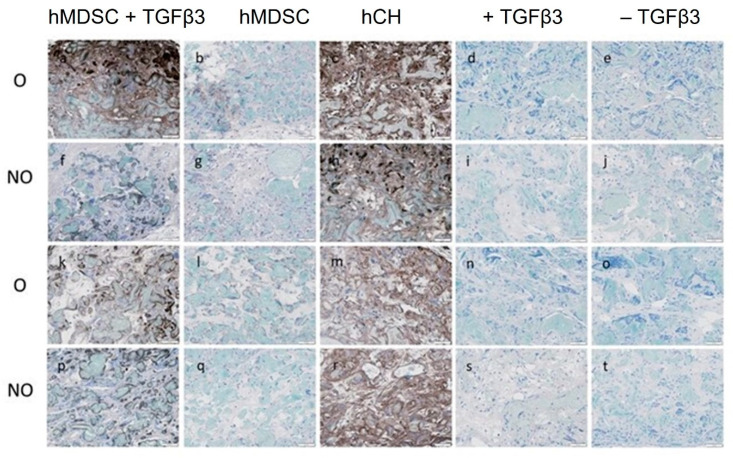
Coll2 expression in O and NO scaffolds in vivo after 4 weeks (**a**–**j**) and 8 weeks (**k**–**t**). Cell-free scaffolds (d,e,i,j,n,o,s,t). NO—untreated, O—ozone-treated, hMDSC—human-muscle-derived stem cells, hCH—human chondrocytes, TGFβ3—transforming growth factor beta-3. Scale bar—50 μm.

Immunohistochemistry staining revealed the presence of Coll2-positive ECM in O and NO scaffolds loaded with TGF-β3 and hMDSCs after 4 and 8 weeks post-implantation. O and NO scaffold–hCH constructs also demonstrated obvious Coll2-positive matrix formation at any time. No Coll2-positive matrix formation was detected in the control group of O and NO cell-free scaffolds (Figure 7).

## 4. Discussion

Due to the complexity of the tissue and inability to self-repair, cartilage has been a complex issue in tissue engineering. In our study, we present an ozone-treated, TGF-β3-loaded scaffold with seeded hMDSCs that demonstrated differentiation into chondrocytes in in vitro experiments and a potential to induce formation of cartilage-like tissue in in vivo SCID mice.

Various scaffolds are used in articular cartilage tissue engineering. Electrospinning is a widely adopted technique using electrostatic forces forming scaffolds from biocompatible material [16]. In our research, we used PCL-derived electrospun scaffolds. The electrospinning was chosen due to the possibility of easily controlling process parameters and parameters of solution, such as viscosity, conductivity, and concentration. Moreover, electrospun scaffolds can easily form ECM structures with interconnecting pores with a high potential to improve formation of different tissues, including cartilage. The fibers can be easily formed in an anisotropic or isotropic direction, thus providing the best close to the native environment for cartilage formation [30,31]. For the electrospinning, we chose PCL as one of the best materials most widely used for tissue, including cartilage regeneration [15,21,32]. According to Garrigues et al., electrospun PCL scaffold demonstrated increased production of collagen type II and sulfated GAGs in 28 d after culturing adipose-derived stem cells [33]. Khatami et al. showed chondrogenesis of MSC on electrospun PCL fibers with higher cell populations and ECM deposition shown by Moura et al. [34,35]. PCL provides various advantages for tissue engineering, but it also has several drawbacks, including limited hydrophilicity and a lack of functional groups required for growth factor incorporation [36,37]. We used hMDSCs for our research; thus, a specific environment including additional protein was important to achieve improved differentiation into chondrocytes. hMDSCs are already widely used in chondrocyte regeneration as they are easily stimulated to form cartilage in vitro as well as in vivo under specific conditions [38,39,40]. Considering the properties of PCL, scaffolds were first treated with ozone (O_3_). O_3_-treated scaffolds may exhibit increased hydrophilic properties [41], which in turn improves cell adhesion and proliferation [42,43]. We observed that electrospun PCL scaffolds provided a proper environment for chondrocytes and hMDSCs. Both cells showed improved proliferation, however, hMDSC demonstrated significantly better growth compared to hCH on O_3_-treated as well as on NO scaffold. Moreover, we were able to observe cell penetration within the scaffold through all the time points, meaning that pore size and microenvironment was suitable not only to grow but to migrate and populate the whole scaffold. Previous studies noted that scaffold pore size and fiber diameter is highly important for cell population and communication [25,44]. High porous scaffolds improve new tissue development and facilitate effective exchange of nutrients and metabolic waste. However, in cartilage regeneration, such factors as scaffolds’ mechanical strength are crucial. Our previous study demonstrated good mechanical and other properties of this electrospun PCL scaffold [27]. Different proteins can facilitate hMDSC differentiation as well as subsequent chondrocyte growth. BMPs as well as FGFs have been shown to orchestrate differentiation into chondrocytes [11,22,45,46]. The TGF-β family displays high potential to induce MSC differentiation into the cartilage tissue [47,48,49,50]. Less is known of their role in hMDSCs differentiation. TGF-β3 is used in MSC application for cartilage regeneration [51] and therefore was selected to be loaded on our scaffolds. As expected, PCL scaffold functionalization with ozone bound a higher amount of TGF-β3 compared to untreated scaffolds. However, it was still unclear if O_3_ treatment is superior, as both scaffolds continuously released close to identical amounts of protein. Nonetheless, in vitro studies demonstrated that O_3_-treatedprotein-loaded scaffolds showed lower amounts of released TGF-β3. This may have been due to the hMDSCs being better adhered to the scaffold surface and more TGF-β3 being bonded to the receptors of the cell, resulting in lower detection of TGF-β3 in the tested cell medium. Yet, no data have suggested the presence of TGF-β3 receptors on the hMDSC membrane. However, the influence of TGF-β3 in the multi-lineage differentiation of stem cells has been observed [52]. Moreover, there is a possibility that better cell adherence led to the better proliferation and higher demand for TGF-β3 regarding higher differentiation into chondrocytes. As expected, higher proliferation was determined on O_3_-treated PCL scaffolds from day 14 compared with non-ozonated ones. These data demonstrate that ozonated scaffolds provide better cell growth conditions for hMDSC. Furthermore, slightly lower expression of Coll2 on day 21, and day 28 by non-ozonated scaffolds clarifies that ozonated surfaces of the scaffold increase hMDSC survival rate and result in better differentiation towards the chondrocytes.

To explore the feasibility of cartilage-like tissue formation in vivo, functionalized scaffold–cell constructs were implanted subcutaneously in SCID/nude mice. Such a strategy is commonly used to test innovative biomaterials with or without supplementation of bioactive molecules that could drive donor or host cells towards chondrogenic lineage [53,54,55]. Safranin O stain indicating deposition of sGAG and Coll2 expression by immunohistochemistry in extracellular matrix of neocartilage are frequently used to justify chondrogenic differentiation of stem cells in vitro and in vivo [56,57]. Our results from our in vivo study demonstrated more pronounced Safranin-O-positive matrix formation on O_3_-treated scaffolds containing TGF-β3 and hMDSCs compared to NO scaffolds at 4 and 8 weeks. At 4 weeks, it was superior compared to both O and NO scaffolds containing hCH. These results suggest that O_3_-treated, TGF-β3 loaded scaffolds provide the proper environment for hMDSCs to undergo chondrogenic differentiation in vivo. In addition, Coll2 expression also confirmed enhanced chondrogenesis on O_3_-treated and NO scaffolds containing TGF-β3 and hMDSC compared to hMDSC-populated scaffolds without TGF-β3 or cell-free scaffolds with or without TGF-β3. It has been observed that expression of chondrogenesis-specific protein was comparable to O and NO scaffolds containing hCH. These findings support other studies indicating that combined (stem cell and protein) delivery systems display a synergistic effect, thereby initiating cartilage regeneration cascade both in vitro and in vivo [53,58,59].

In summary, our study showed electrospun PCL scaffold’s functionalization via ozone treatment and loading with TGF-β3-stimulated hMDSC differentiation into a cartilage tissue in vitro and in an in vivo animal model. This is the first articular cartilage regeneration study demonstrating a promising result of the combination of three different factors, namely, treatment with ozone, TGF-β3 loading, and hMDSCs. However, it has some limitations. Only some concentrations of protein have been tested; thus, different concentrations of growth factor should be evaluated with a potential lowest effective dosage. This is important to optimize economic cost and to prevent potential side effects of residual protein on surrounding tissues after implantation. Moreover, TGF-β3 binding time could be further optimized with the most desirable effect on stem cell differentiation. Nevertheless, in vitro experiments are not always 100% corresponding to the in vivo studies, as the cells are in an artificial environment (e.g., in plastic wells). Thus, further in vivo animal studies are necessary to confirm the functional and mechanical capacity of this scaffold–stem cell construct.
